# Analysis of the sagittal root angle and its correlation with hard and soft tissue indices in anterior teeth for immediate implant evaluation: a retrospective study

**DOI:** 10.1186/s12903-021-01848-x

**Published:** 2021-10-04

**Authors:** Mengru Shi, Xiaoshuang Wang, Peisheng Zeng, Haiwen Liu, Zhuohong Gong, Yixiong Lin, Zhipeng Li, Zetao Chen, Zhuofan Chen

**Affiliations:** 1 Hospital of Stomatology, Guanghua School of Stomatology, Sun Yat-sen University and Guangdong Provincial Key Laboratory of Stomatology, Guangzhou, China; 2 Guangdong Research Center for Dental and Cranial Rehabilitation and Material Engineering, Guangzhou, China

**Keywords:** Immediate implant placement, Sagittal angle, Root inclination, Alveolar bone, Maxillary anterior tooth

## Abstract

**Background:**

To assess the root angle characteristics of maxillary incisors, and to analyze the relationship between the root angle and other implant-related anatomical indices to use the sagittal root angle as an index for immediate implant evaluation and design.

**Methods:**

A random sample consisting of 400 cone-beam computed tomography (CBCT) images and 65 maxillary plaster models were selected for the present study. CBCT and stereolithography (STL) scan images were imported as DICOM files into coDiagnostiX software for matching the hard and soft tissue. The angle between the long axis of the anterior tooth and the corresponding alveolar bone and implant-related hard and soft tissue indices were measured in the sagittal section. Descriptive statistics, frequency analysis, multi-level comparisons, and correlation analyses were performed.

**Results:**

The average sagittal root angles were 15° at the central incisor and 19° at the lateral incisor. The root angle in males was significantly larger than that in females, and increased with age. The largest angle, 22.35°, was found in the lateral incisors of the oldest (> 50 years old) male group. The root angle was found to correlate with coronal buccal bone thickness, coronal palatal bone thickness, apical buccal bone thickness, palatal bone thickness, and the below apex bone thickness.

**Conclusions:**

The sagittal root angle could reflect the distribution of other implant-related anatomical indices, which may provide additional reference for the evaluation of immediate implant placement.

**Supplementary Information:**

The online version contains supplementary material available at 10.1186/s12903-021-01848-x.

## Background

Immediate implant placement, especially in the anterior region of the maxilla, is a delicate decision, and some factors need to be taken into consideration, such as anatomical characteristics, surgical techniques, and prosthodontics design [1]. It is important to conduct thorough assessments before making implant treatment plans to obtain ideal and predictable outcomes. The anatomical factors that have gained the most attention include alveolar bone wall thickness and gingiva phenotype, which are critical for determining implant position design and for ensuring long-term esthetic outcomes [2, 3]. The sagittal root angle, which corresponds to the alveolar bone characteristics, plays a key role in determining implant position [3–5].

The sagittal root angle, which measures the long axis as an immediate index for implant placement, is mainly used as the concept of restoration-orientation [4, 6, 7]. The ideal implant axis is proposed to correspond to the axis of the contralateral and adjacent tooth inside the alveolar bone, and the implant is inclined to palatal [8, 9]. Limited by insufficient bone dimensions around the tooth root, especially on the labial side, the root apex, and a thin gingiva phenotype, it is hard to place an implant following the axis of the contralateral corresponding tooth, as bone fenestration and dehiscence may occur [10–12]. Thus, a pre-implantation evaluation of the root angle, accounting for the characteristics of the surrounding implant-related hard and soft tissues, is crucial for planning an immediate implant placement.

Previous studies have reported that the sagittal root angle is also closely related to the surrounding implant-related hard and soft tissues [13–15], which implies that the root angle is not simply an implant inclination indicator, but may have many more uses. However, insufficient understanding of the relationship between the root angle and other anatomical indices limits its clinical applications. Sagittal root angle has not been used routinely as a vital anatomical index for pre-implantation evaluation and design. It is of great clinical significance to elucidate the relationship between the sagittal root angle and other immediate implant-related hard and soft tissue indices, so that we can use the root angle as an index to optimize the evaluation and design of the immediate implant process. Therefore, in this manuscript, we explored the root angle characteristics of maxillary incisors, and analyzed the relationship between the sagittal root angle and other implant-related alveolar bone, tooth, and gingiva indices around the root.

## Methods

### Data collection and study design

The protocol for the present study was approved by the Ethics Committee of the Sun Yat-sen University Hospital. Being a retrospective study, the need of informed consent was waived by the Ethics Committee of the Sun Yat-sen University Hospital (KQEC-2020-29). The cone beam computed tomography (CBCT) data and plaster models used for scanning were obtained from databases at the Departments of Oral Radiology and Oral Implantology, Hospital of Stomatology, Guanghua School of Stomatology, Sun Yat-sen University from October 1, 2019 to July 10, 2010. For the analysis of the sagittal root angle characteristics in different teeth, age, and gender groups in the study population. The data were taken from a representative sample of patients who underwent CBCT imaging and had plaster models for various indications. Data including age and gender were also collected with the CBCT scans selected for measurements.

#### Sample size

In the present study, we calculated the minimum sample size [16] with 95% confidence level (α = 0.05) and 80% power of test (β = 0.2) using the website https://sample-size.net/. The total group size required was 196 subjects, calculated using the formula N = AB / (E / S).

#### Inclusion criteria

(1) CBCT images of subjects aged 18 years or older; (2) CBCT images including four natural teeth in the region of interest (from the right lateral to the left lateral incisors) that were of acceptable quality for performing the measurements; (3) CBCT images were obtained using the same machine and general technical settings; (4) Maxillary plaster models with intact labial gingiva in the region of interest (from right lateral to left lateral incisors) were of acceptable quality for performing the measurements [17].

#### Exclusion criteria

(1) CBCT images indicating severe alveolar bone resorption, severe periodontitis or crowding, and infectious pathology; (2) CBCT images with distorted or scatter artifact; (3) CBCT images showing orthodontic treatments or restorations; (4) CBCT images indicating evident root resorption, root fracture apical resection, and/or periapical lesions [18]; (5) Maxillary plaster models with damage and/or evident bubbles in the region of interest (from the right lateral to the left lateral incisors).

### Acquisition of CBCT images and stereolithography (STL) images

CBCT images (NewTom VG; QR s.r.l., Verona, Italy) were acquired with a voxel size of 0.3 mm, exported in DICOM multi-file format, and imported into coDiagnostiX(version 9.12; Dentalwings, Montreal, Canada) to evaluate the central sagittal section of the anterior teeth.

Plaster molds were imaged using an intraoral scanner (CS3600, Carestream Dental, Rochester, NY, USA), following the manufacturer's instructions. The scan images were exported in STL format, and imported into coDiagnostiX for matching with the CBCT images from the same individual and evaluation of the central sagittal section of the anterior teeth [19].

### Data measurement

**Fig. 1:**
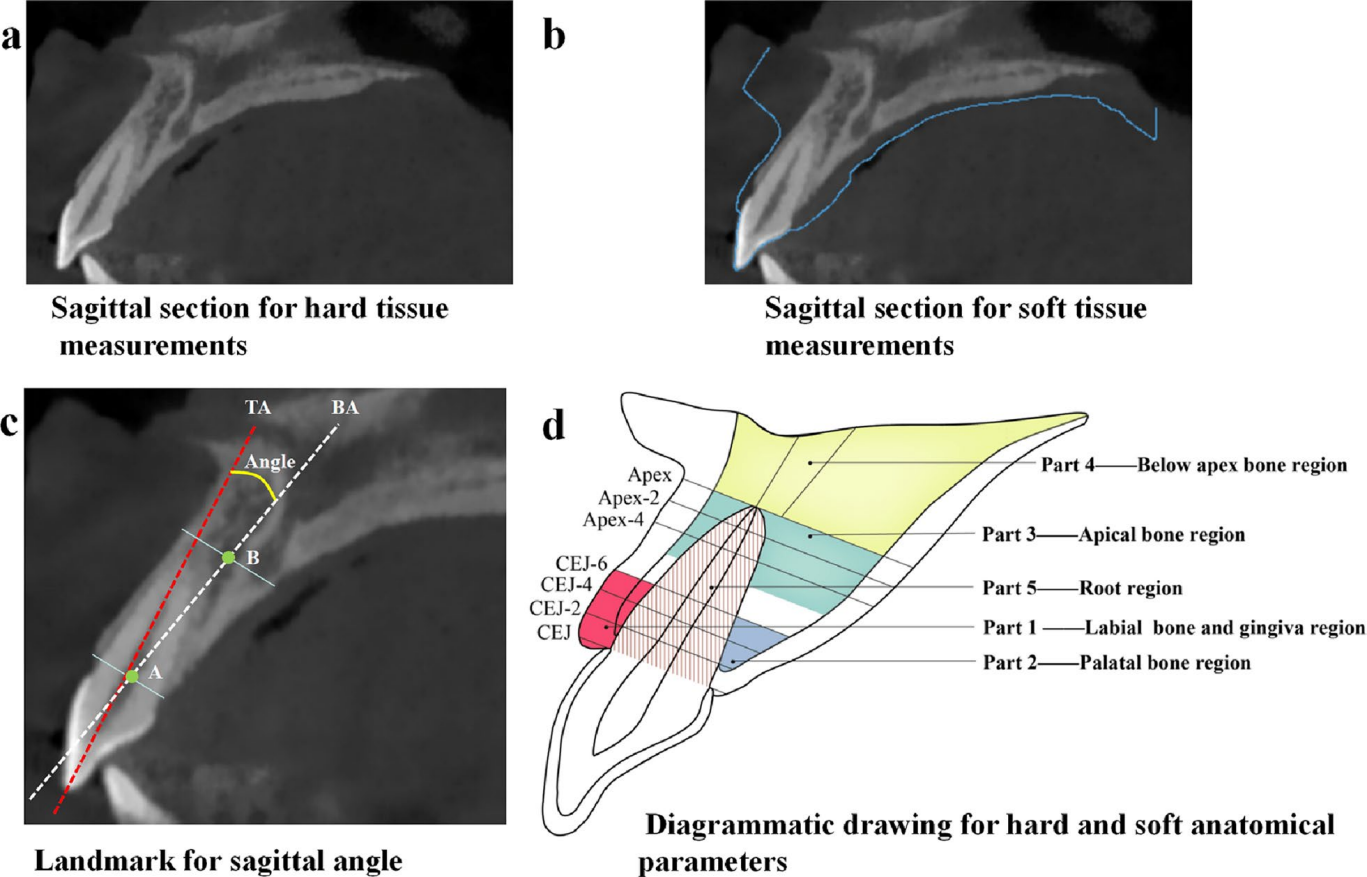
Standardized sagittal section images for **a** hard tissue and **b** soft tissue measurements**. c** Landmarks for the sagittal root angle, between the long axis of tooth and the long axis of the alveolar bone. The red line shows the tooth axis (TA) in the sagittal plane, crossing the incisor and apical points. The white line shows the corresponding alveolar bone axis (BA). To determine BA, a line was drawn joining the palatal and buccal bone crests, with the midpoint defined as A. Another line was drawn parallel to this line, 2 mm apical to the root apex, the midpoint of the labial and palatal surfaces was defined as B. BA is a straight line crossing the two midpoints, A and B. The angle between TA and BA (TA-BA) was measured in degrees. **d** Dimensions of hard tissue and thickness of labial gingiva measurements. Part 1 was buccal bone and gingiva dimensions, including the thickness of labial gingiva and bone at the CEJ and at levels 2, 4, and 6 mm apical to the CEJ (C-2, C-4, C-6 labial gingiva thickness and C-2, C-4, C-6 buccal bone thickness). Part 2 represented palatal bone dimensions, including the thickness of the palatal bone wall at levels 2, 4, and 6 mm apical to the CEJ (C-2, C-4, C-6 palatal bone thickness). Part 3 represented apical bone dimensions, including the thickness of the labial and palatal bone walls around the apex, measured at the apex and at levels 2 and 4 mm coronal to the apex, respectively (R-0, R-2, R-4 buccal bone thickness and R-0, R-2, R-4 palatal bone thickness). Part 4 represented bone dimensions below the apex, including the length of the root apex to the alveolar palatal plane, measured along the long axis of the anterior tooth (TA-Apex to Palate) and the corresponding alveolar bone axis (BA-Apex to Palate). Part 5 represented root dimensions, including the root diameters measured at levels 2, 4, and 6 mm apical to the CEJ, 2, 4 mm coronal to the apex (C-0, C-2, C-4, C-6, R-2, R-4 root diameter)

Standardized protocols were used to obtain the sagittal sections to be used for measurement (Additional file 1: Fig. S1). Sagittal sections of four anterior teeth were saved as TIFF files to be used for hard and soft tissue measurements (Fig. 1a, b). STL formatted scan images were imported into coDiagnostiX™ and matched with the CBCT images from the same individual, after which the central sagittal sections of the anterior teeth were captured. This experiment has developed a standardized measurement process to obtain pictures and measurement data, and try to avoid measurement bias in the measurement process [19, 20].

All sagittal section images were processed using Adobe Illustrator software (version 4.0, Adobe Systems Inc, California, USA). The sagittal root angle was defined as the angle between the long axis of the anterior tooth and the long axis of the corresponding alveolar bone in the sagittal plane (Fig. 1c). Other implant-related hard and soft anatomical indices were divided into five parts, based on their clinical significance (Fig. 1d).

*Part 1. Buccal bone and gingiva dimensions* are presented in the red region, and are related to possible bone fenestration or perforation at the labial side, as well as esthetic outcomes. The thickness of the labial gingiva and bone at the cemento-enamel junction (CEJ) and at levels 2, 4, and 6 mm apical to the CEJ levels were measured.

*Part 2. Palatal bone dimensions* are presented in the blue region, and may provide an alternative bone for implant placement. The thickness of the palatal bone wall at levels 2, 4, and 6 mm apical to the CEJ were measured.

*Part 3. Apical bone dimensions* are presented in the green region, and are closely related to the primary stability of the implants. The thickness of the labial and palatal bone wall around the apex was measured at the apex and at levels 2 mm and 4 mm coronal to the apex, respectively.

*Part 4. The bone dimensions under the root apex* are presented in the yellow region, and can be used to determine the embedding depth of the implant. The length from the root apex to the alveolar palatal plane was measured along the long axis of the anterior tooth and the corresponding alveolar bone.

*Part 5. Root dimensions* are presented in the brown region, and have a close relationship with the implant diameter and placement gap. The root diameters were measured at levels 2, 4, and 6 mm apical to the CEJ, and 2 mm and 4 mm coronal to the apex.

Refer to Additional file 2: Fig. S2 for detailed measurement steps.

### Statistical analysis

Statistical analysis was performed using the SPSS (Version 20; Inc., Chicago, IL, USA) standard statistical package. Levene’s test was used to evaluate the variance equality of the samples. For two independent samples with variance equality, Student’s t-test was used. Otherwise, the non-parametric Mann–Whitney test was used. The parametric ANOVA test was used to compare three or more independent samples. Otherwise, the non-parametric Kruskal–Wallis test was used. Karl Pearson correlation coefficients (R) were used to assess the relationships between sagittal root angle and bone thickness, root width, and gingiva thickness. *P* < 0.05 was considered statistically significant. For the analysis of the correlation between root angle and other hard and soft tissue indices [21], the total group size required was 92 subjects, calculated using the formula N = [(Z_α_ + Z_β_)/C]^2^ + 3.

## Results

### Sagittal root angle characteristics in the study population

Imaging data from 400 patients, totaling 1600 teeth (4 teeth from each patient, including the right and left central and lateral incisors), and 65 maxillary plaster models, including 130 anterior teeth, were selected for the present study. The average age was 35.88 years (range, 19–80 years), and of the 400 patients, 172 (43%) were male and 228 (57%) were female.

**Fig. 2:**
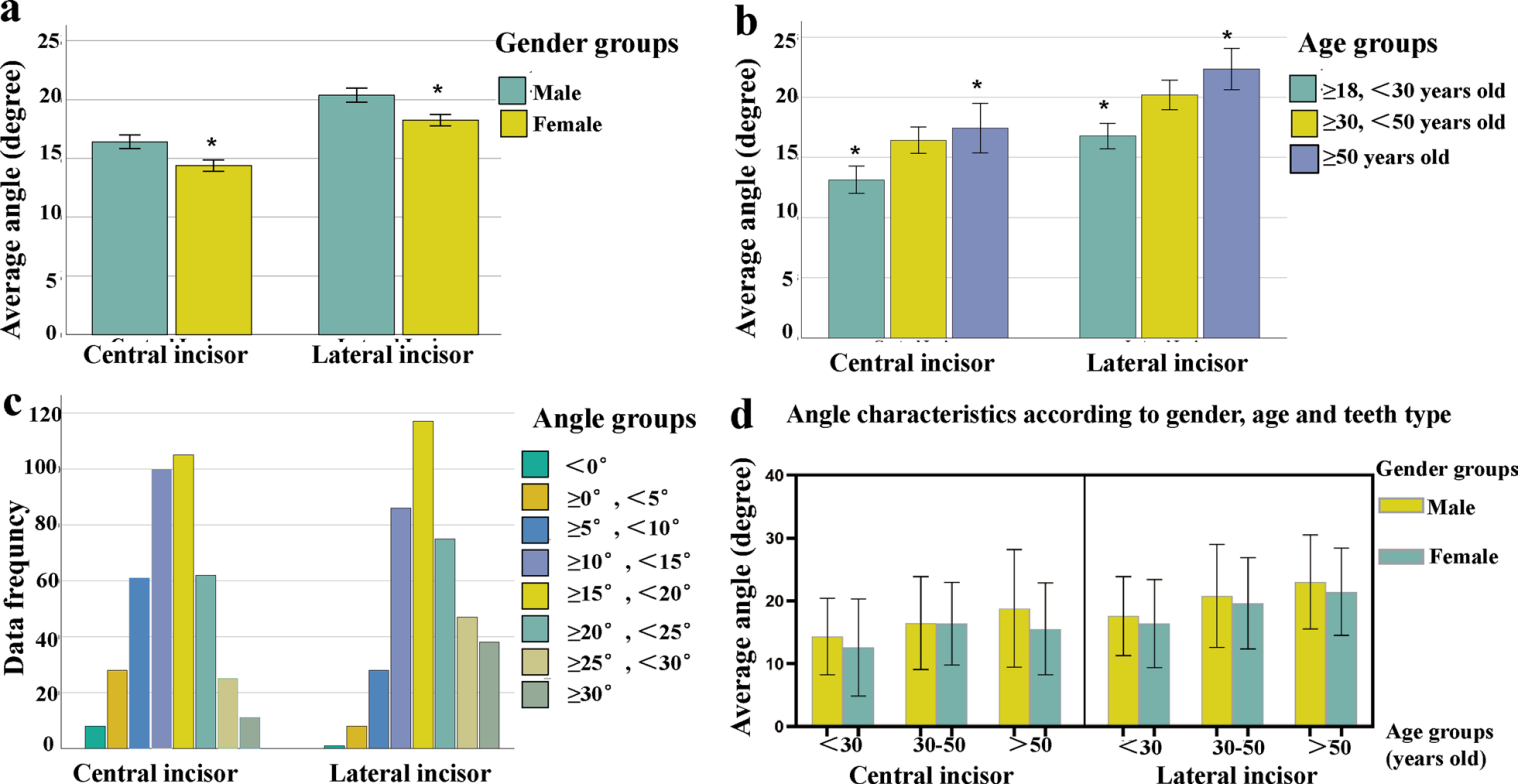
Bar charts represent the mean and standard deviation (SD) of different measurements. **a** Angle characteristics in male and female groups (mean ± SD). **b** Angle characteristics in 18–30, 30–50, > 50 years-old age groups (mean ± SD). **c** Angle distribution in < 0°, 0°–5°, 5°–10°, 10°–15°, 15°–20°, 20°–25°, 25°–30°, > 30° groups. **d** Angle characteristics in different sex and age groups (mean ± SD)

The angle of the right central incisor was 15.24° ± 7.61°, 14.99° ± 7.08° for the left central incisor, 19.15° ± 7.56° for the right lateral incisor, and 19.24° ± 7.61° for the left lateral incisor. The results indicated significant differences between the central and lateral incisors on both the right and left sides (*P* < 0.01). There was no significant difference between the two central incisors or the two lateral incisors (Table 1). Based on these results, only the right central and right lateral incisors were chosen for subsequent research. To better understand the distribution characteristics of the sagittal root angle, the frequency distribution was analyzed (Table 2; Fig. 2c). The angle was primarily distributed between 15 and 20° in both the central and lateral incisors.

**Table 1:** Angle characteristics according to teeth location and teeth type (mean ± SD)

Teeth location	Angle		Statistic test (*P* value)
	Central incisor	Lateral incisor	
Right side	15.24 ± 7.61	19.15 ± 7.56	*P* < 0.01
Left side	14.99 ± 7.08	19.24 ± 7.61	*P* < 0.01
Statistic test (*P* value)	*P* = 0.624	*P* = 0.881

**Table 2:** Angle characteristics according to angle group and teeth type (mean ± SD)

Angle group	Angle (n, %row)		
	Center incisor	Lateral incisor	Overall
< 0°	− 3.87 ± 2.90 (8, 2%)	− 0.12 (1, 0.25%)	− 3.45 ± 2.99 (9, 1.12%)
≥ 0° < 5°	3.33 ± 1.20 (28, 7%)	2.73 ± 1.33 (8, 2%)	3.20 ± 1.24 (36, 4.5%)
≥ 5° < 10°	7.87 ± 1.36 (61, 15.25%)	8.41 ± 1.35 (28, 7%)	8.04 ± 1.37 (89, 11.13%)
≥ 10° < 15°	12.66 ± 1.41 (100, 25%)	13.03 ± 1.46 (86, 21.5%)	12.83 ± 1.44 (186, 23.25%)
≥ 15° < 20°	17.66 ± 1.38 (105, 26.25%)	17.30 ± 1.44 (117, 29.25%)	17.47 ± 1.42 (222, 27.75%)
≥ 20° < 25°	22.28 ± 1.43 (62, 15.5%)	22.53 ± 1.41 (75, 18.75%)	22.42 ± 1.42 (137, 17.13%)
≥ 25° < 30°	27.03 ± 1.22 (25, 6.25%)	27.24 ± 1.36 (47, 11.75%)	27.17 ± 1.31 (72, 9%)
≥ 30°	34.35 ± 5.87 (11, 2.75%)	33.97 ± 3.50 (38, 9.5%)	34.05 ± 4.08 (49, 6.13%)
Overall	15.24 ± 7.62 (400, 100%)	19.16 ± 7.56 (400, 100%)	17.20 ± 7.84 (800, 100%)

*n* indicates no. of involved teeth

#### Angle characteristics according to age group

The sample population was divided into three groups according to age: ≥ 18 < 30 years, ≥ 30 < 50 years, and ≥ 50 years. The average sagittal root angle for each group was as follows: 13.14° ± 7.30°, 16.41° ± 6.96°, and 17.45° ± 8.65° for the central incisor, and 16.79° ± 6.81°, 20.16° ± 7.72°, and 22.35° ± 7.25° for the lateral incisor, respectively (Table 3). Significant differences were found between the three age groups for both the central and lateral incisors (*P* < 0.01), as seen in Fig. 2b.

**Table 3:** Angle characteristics according to age group (mean ± SD)

Age group	N (%row)	Angle (n, %row)		
		Central incisor	Lateral incisor	Overall
≥ 18 < 30	330 (41.25)	13.14 ± 7.30	16.79 ± 6.81	14.96 ± 7.28
≥ 30 < 50	328 (41)	16.41 ± 6.96	20.16 ± 7.72	18.28 ± 7.57
≥ 50	142 (17.75)	17.45 ± 8.65	22.35 ± 7.25	19.90 ± 8.32
Statistic test (*P* value)		*P* < 0.01	*P* < 0.01	*P* < 0.01

*N* indicates no. of involved individuals

#### Angle characteristics according to gender

The sagittal root angles in males and females were 16.40° ± 7.70° and 14.37° ± 7.46° for the central incisor, 20.36° ± 7.73°, and 18.25° ± 7.33° for the lateral incisor, respectively (Table 4). Significant differences were found between males and females for both the central and lateral incisors (*P* < 0.01), as seen in Fig. 2a.

**Table 4:** Angle characteristics according to gender (mean ± SD)

Gender	N (%row)	Angle (n, %row)		
		Central incisor	Lateral incisor	Overall
Male	344 (43%)	16.40 ± 7.70	20.36 ± 7.73	18.38 ± 7.95
Female	456 (57%)	14.37 ± 7.46	18.25 ± 7.33	16.31 ± 7.63
Statistic test (*P* value)		*P* < 0.01	*P* < 0.01	*P* < 0.01

*N* indicates no. of involved individuals

#### Angle characteristics according to age and gender

The sagittal root angles for central and lateral incisors in males and females of different age groups are presented in Fig. 2d. Overall, the lateral incisor in males > 50 years of age had the largest angle, while the central incisor in females 18–30 years of age had the smallest angle.

### The correlation of sagittal root angle with other implant-related hard and soft tissue indices

The correlation analysis results are presented as *P-*values and coefficient of correlation *R*-values, as seen in Table 5.

**Table 5:** The correlation between angle and other implant-related bone, gingiva and teeth indicators in anterior teeth

Type	Parameter	n	*P* value	*R* value
Buccal bone and gingival dimension	C-2 buccal bone thickness	800	0.01	− 0.113
	C-4 buccal bone thickness	800	0.168	− 0.049
	C-6 buccal bone thickness	800	0.017	− 0.085
	C-2 Labial gingival thickness	130	.146	− 0.128
	C-4 Labial gingival thickness	130	.353	− 0.082
	C-6 Labial gingival thickness	130	.447	− 0.067
Palatal bone dimension	C-2 Palatal bone thickness	800	0.533	0.022
	C-4 Palatal bone thickness	800	< 0.01	0.284
	C-6 Palatal bone thickness	800	< 0.01	0.476
Apical bone dimension	R-0 buccal bone thickness	800	< 0.01	− 0.501
	R-2 buccal bone thickness	800	< 0.01	− 0.247
	R-4 buccal bone thickness	800	< 0.01	− 0.102
	R-0 Palatal bone thickness	800	< 0.01	0.604
	R-2 Palatal bone thickness	800	< 0.01	0.517
	R-4 Palatal bone thickness	800	< 0.01	0.422
Alveolar bone dimension	TA-Apex to Palate	800	< 0.01	− 0.467
	BA-Apex to Palate	800	< 0.01	0.192
Root dimension	C-0 root diameter	800	< 0.01	− 0.137
	C-2 Root diameter	800	0.892	− 0.005
	C-4 Root diameter	800	0.632	− 0.017
	C-6 Root diameter	800	0.111	− 0.056
	R-2 Root diameter	800	0.295	0.039
	R-4 Root diameter	800	0.883	0.005

*n* indicates no. of involved teeth

#### Part 1. Buccal bone and gingiva dimension

The thickness of the labial gingiva at the CEJ and at levels 2, 4, and 6 mm apical to the CEJ had no correlation with the sagittal root angle (*P* > 0.05), while the thickness of the buccal bone wall at levels 2 and 6 mm apical to the CEJ had a negative correlation.

#### Part 2. Palatal bone dimension

The thickness of the palatal bone wall at levels 4 and 6 mm apical to the CEJ have a significant positive correlation with the sagittal root angle.

#### Part 3. Apical bone dimension

The thickness of the buccal bone wall at the apex and at levels 2 mm and 4 mm coronal to the apex have significant negative correlations with the sagittal root angle. The thickness of the palatal bone wall at the apex and at levels 2 mm and 4 mm coronal to the apex have a significant positive correlation with the angle.

#### Part 4. Under root apex bone dimension

The length from the root apex to the alveolar palatal plane along the long axis of the anterior tooth had a significant negative correlation with the sagittal root angle. However, the length from the root apex to the alveolar palatal plane along the long axis of the bone  has a significant positive correlation with the angle.

#### Part 5. Root dimension

The root diameter at levels 2, 4, and 6 mm apical to the CEJ, and at levels 2 mm and 4 mm coronal to the apex have no correlation with the sagittal root angle (*P* > 0.05).

### Scatter diagram and regression equations for sagittal root angle with correlated indices

**Fig. 3:**
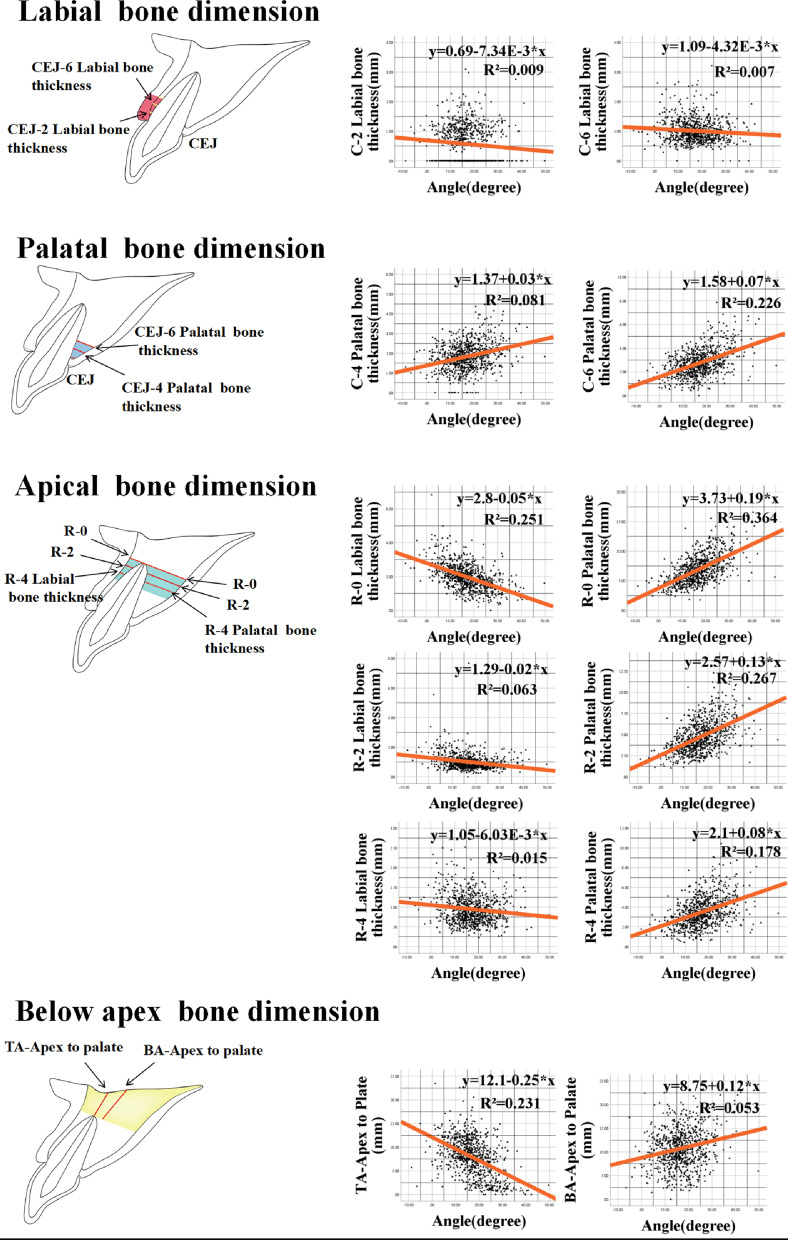
Line tables represent 5 parts of implant-related hard and soft tissue indices in different angle groups. The dotted lines indicate the no correlation parameter (*P* > 0.05), while the solid lines indicate the correlation parameter (*P* < 0.05)

A scatter diagram and regression equation were created for each pair of sagittal root angles and correlated indices, respectively (Fig. 3). From the regression analysis, only five indices had regression equations with goodness of fit values of R^2^ > 0.1. For the thickness of the palatal bone 6 mm apical to the CEJ, Y1 = 1.58 + 0.07 × angle (R^2^ = 0.226). For the thickness of the buccal bone at the apex, Y2 = 3.73 + 0.19 × angle (R^2^ = 0.364). For the thickness of the buccal bone 2 mm coronal to the apex, Y3 = 2.57 + 0.13 × angle (R^2^ = 0.267). For the buccal bone thickness 2 mm coronal to the apex, Y4 = 2.1 + 0.08 × angle (R^2^ = 0.178). For the length from the apex to the palatal plane along the axis of the tooth, Y5 = 12.1 + 0.25 × angle (R^2^ = 0.231).

### Alveolar bone, tooth and gingiva distribution characteristics in different angle groups

**Fig. 4:**
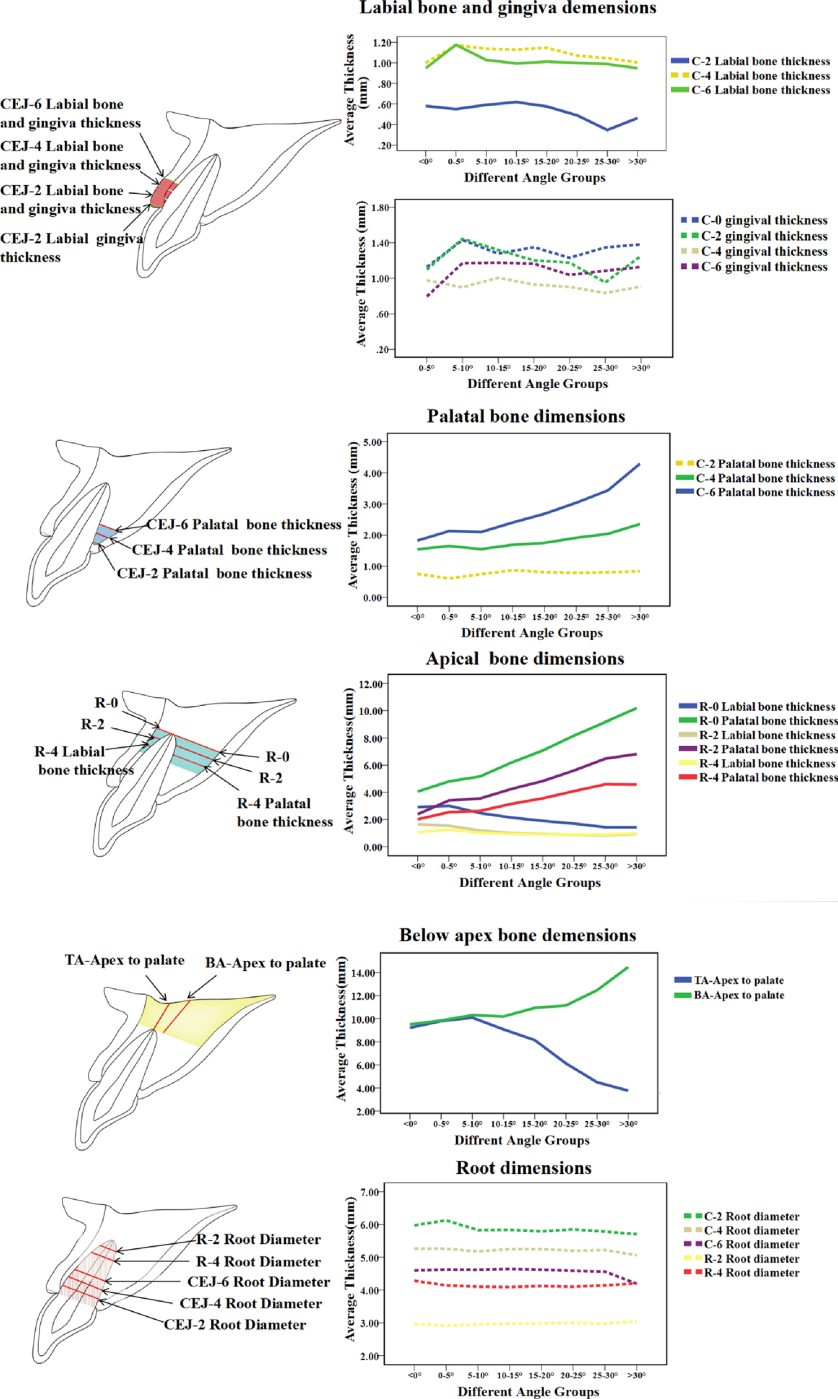
Linear regression plots for sagittal root angle and other correlated implant-related anatomic parameters in anterior teeth

To better understand how the sagittal root angle influenced the correlative index, the angle was divided into 8 groups: < 0°, ≥ 0° < 5°, ≥ 5° < 10°, ≥ 10° < 15°, ≥ 15° < 20°, ≥ 20° < 25°, and ≥ 30°, which were then used to create a line chart for the root angle and other related anatomical indices (Fig. 4). The average thickness of these angle-correlated bone indices for each angle group is displayed in Table 6. For coronal buccal bone and gingiva dimensions, as the root angle increased, the thickness of buccal bone decreased, without a regular trend. When the root angle was > 25°, the coronal buccal bone thicknesses at levels 2, 4, and 6 mm apical to the CEJ were < 1 mm. For coronal palatal bone dimensions, as the root angle increased, the bone thickness increased, with an obvious trend. For all angle groups, the thickness of the coronal palatal bone was > 1 mm. When the root angle was < 0°, the thickness of the palatal bone was < 2 mm. For the apical bone dimensions, as the root angle increased, the thickness of buccal bone decreased, with an obvious trend, while the palatal bone thickness at the corresponding levels increased steadily. When the root angle was > 10°, the buccal bone thickness was < 1 mm. For the bone dimensions under the root apex, as the root angle increased, the length from the apex to the palatal plane at the axis of the tooth decreased, with an obvious trend, while the length from the apex to the palatal plane at the axis of the bone increased, with an obvious trend. When the angle was > 25°, the length from the apex to the palatal plane along the long axis of the tooth was < 6 mm.

**Table 6:** Angle correlated indicators in anterior teeth in different angle groups

Angle groups	C-2 Buccal bone thickness	C-6 Buccal bone thickness	C-4 Palatal bone thickness	C-6 Palatal bone thickness	R-0 Buccal bone thickness	R-2 Buccal bone thickness
< 0°	0.86 ± 0.45	0.86 ± 0.27	1.50 ± 0.54	1.70 ± 0.64	3.02 ± 0.71	1.53 ± 0.55
≥ 0° < 5°	0.51 ± 0.58	1.20 ± 0.54	1.61 ± 0.67	2.03 ± 0.91	2.98 ± 1.01	1.57 ± 0.95
≥ 5° < 10°	0.59 ± 0.58	1.00 ± 0.44	1.64 ± 0.62	2.25 ± 0.82	2.45 ± 0.76	1.19 ± 0.61
≥ 10° < 15°	0.65 ± 0.59	1.01 ± 0.36	1.68 ± 0.63	2.39 ± 0.82	2.12 ± 0.59	0.98 ± 0.38
≥ 15° < 20°	0.60 ± 0.67	1.03 ± 0.39	1.76 ± 0.69	2.66 ± 0.88	1.92 ± 0.66	0.94 ± 0.39
≥ 20° < 25°	0.50 ± 0.61	1.00 ± 0.39	2.02 ± 0.80	3.13 ± 1.15	1.70 ± 0.56	0.89 ± 0.34
≥ 25° < 30°	0.37 ± 0.54	0.95 ± 0.34	2.16 ± 0.75	3.46 ± 1.19	1.41 ± 0.54	0.81 ± 0.24
≥ 30°	0.52 ± 0.69	0.94 ± 0.40	2.41 ± 1.04	4.29 ± 1.52	1.40 ± 0.65	0.97 ± 0.41
Overall	0.56 ± 0.62	1.01 ± 0.39	1.84 ± 0.75	2.77 ± 1.13	1.97 ± 0.76	0.99 ± 0.47

Angle groups	R-4 Buccal bone thickness	R-0 Palatal bone thickness	R-2 Palatal bone thickness	R-4 Palatal bone thickness	TA-Apex to Plate	BA-Apex to Plate
< 0°	1.00 ± 0.34	3.94 ± 1.04	2.16 ± 0.71	1.96 ± 0.78	8.44 ± 4.48	8.04 ± 4.39
≥ 0° < 5°	1.29 ± 0.56	4.43 ± 1.29	3.09 ± 1.03	2.32 ± 0.87	9.89 ± 4.22	9.58 ± 4.33
≥ 5° < 10°	1.02 ± 0.46	5.12 ± 1.46	3.55 ± 1.25	2.67 ± 1.15	10.13 ± 2.85	10.42 ± 3.43
≥ 10° < 15°	0.92 ± 0.34	6.05 ± 1.69	4.18 ± 1.46	3.10 ± 1.20	9.38 ± 3.14	10.09 ± 3.88
≥ 15° < 20°	0.93 ± 0.37	6.86 ± 1.85	4.72 ± 1.53	3.51 ± 1.26	8.31 ± 3.65	10.68 ± 3.66
≥ 20° < 25°	0.89 ± 0.32	8.13 ± 2.30	5.58 ± 2.00	4.11 ± 1.68	6.40 ± 3.62	10.85 ± 4.30
≥ 25° < 30°	0.86 ± 0.30	8.77 ± 2.17	6.20 ± 1.97	4.43 ± 1.59	4.64 ± 3.99	12.17 ± 3.99
≥ 30°	1.01 ± 0.41	10.29 ± 2.74	6.93 ± 2.38	4.73 ± 1.84	2.90 ± 3.02	13.98 ± 4.35
Overall	0.95 ± 0.38	6.93 ± 2.42	4.78 ± 1.94	3.51 ± 1.52	7.85 ± 4.03	10.80 ± 4.04

## Discussion

In the present study we explored the characteristics of the sagittal root angle of maxillary incisors in the study population, and analyzed the correlation between the angle and the dimensions of the alveolar bone, teeth, and gingiva around the root, in an effort to help clinicians better understand and utilize the angle as a clinical index for immediate implant placement design. In the study population, the angle was on average 15° at the central incisor and 19° at the lateral incisor, with the majority ranging 10°–25°. The angle was found to be larger in males than in females, and larger in the older than in the younger group. The sagittal root angle may reflect the distribution of other implant-related anatomical indices. For the correlation of the root angle with other immediate implant-related hard and soft tissue anatomical indices, the smaller angle corresponded to thinner buccal bone and thicker palatal bone around both the CEJ and apex, and more available bone volume under the root apex along the tooth axis, which may provide further indications for immediate implant placement.

### Sagittal root angle characteristics in the study population

There was no significant difference in the sagittal root angle between the right and left central incisors and the right and left lateral incisors, which was consistent with previous studies [10, 22]. The angle of the lateral incisor was found to be nearly 4° larger than that of the central incisor. The angle falls mostly in the range of 10°–25°, and is rarely found in the extreme regions at either end, < 0° or > 30°, for both the central and lateral incisors. This suggests that most of the anterior teeth were inclined in the coronal direction relative to the alveolar bone.

The sagittal root angle revealed a tendency to increase with age, and significant differences were found between different age groups. Alveolar bone changes physiologically with age [23, 24]. The results of the present study show that outward and upward absorption of the alveolar bone caused the teeth to move palatal in relation to the alveolar bone, creating a gradual increase in angle. The angle in males was nearly 2° larger than in females. Combined with age and gender factors of the study population, the lateral incisor had the largest angle, 23.46°, in the oldest male group, and the central incisor had the smallest angle, 12.86°, in the youngest female group.

### Correlation of sagittal root angle with immediate implant-related hard and soft tissue indices

The sagittal root angle was significantly correlated with apical bone wall thickness and bone thickness under the root apex and palatal bone wall (*p* < 0.01), and had a moderate correlation with coronal buccal bone thickness (*p* < 0.05). No correlation was found between labial gingiva thickness and root diameter at any position (*p* > 0.05). These results revealed that the sagittal root angle may reflect the characteristics of other implant-related anatomical indices, especially the bone distribution around the CEJ, root apex, and under the root apex.

For coronal buccal bone thickness, the thickness at levels 2 and 6 mm apical to the CEJ were negatively correlated with the root angle, but without a regular trend. In regards to esthetics, a minimum labial wall thickness of 2 mm was required to provide adequate soft tissue support and prevent further gingiva recession [25, 26]. A larger root angle corresponded to thinner buccal bone, which was accompanied by higher esthetic risks. The results of the present study indicated that the buccal bone thickness was always < 2 mm, which suggested that CBCT was indispensable to evaluate the buccal bone thickness when planning an immediate implant for possible bone augmentation procedure due to concern for long-term esthetics (Table 6). When the angle was > 25°, the buccal bone thickness at levels 2, 4, and 6 mm apical to the CEJ was < 1 mm, which reflected the increased risk of buccal bone wall defect when placing an implant [27, 28].

For coronal palatal bone wall thickness, the thickness at levels 4 and 6 mm apical to the CEJ was positively correlated with the root angle, without a regular trend. For all angle groups, the thickness of the coronal palatal bone was > 1 mm (Table 6). When the root angle was < 0°, the thickness of the bone wall was < 2 mm, which was relatively thin. Increased root angles reflected thicker palatal bone, and demonstrated that more reserved bone volume could be used to adjust the implant to the proper position [14].

For the apical bone dimension, as the sagittal root angle increased, the thickness of the palatal bone at the apex and at levels 2 mm and 4 mm coronal to the apex increased gradually, while the corresponding labial wall thickness decreased slightly. The apical palatal bone had an average thickness of 3.51–6.93 mm (Table 6), which was typically sufficient for the primary stability of the implant. The apical buccal bone was thin, with an average thickness of 0.94–1.97 mm, which required more attention when placing an implant, in order to protect the intact buccal bone wall. Smaller sagittal root angels were related to thicker buccal bone around the apex, which indicated a better likelihood for sufficient primary stability for immediate implant placement. The results of the present study showed that when the root angle was > 10°, the buccal bone thickness at levels 2 mm and 4 mm coronal to the apex was > 1 mm (Table 6), indicating a higher risk of fenestration and perforation during implant placement.

For the bone dimension under the root apex, as the sagittal root angle increased, the length from the root apex to the alveolar palatal plane along the long axis of the tooth gradually decreased, while the length from the root apex to the alveolar palatal plane along the long axis of the anterior tooth gradually increased. It was thought that in order to ensure sufficient primary implant stability, the implant should be inserted into the socket 4 to 5 mm beyond the root apex [29]. In addition, an extra 1–2 mm thickness of the bone wall would be preferred, in order to avoid perforation and to protect any important adjacent anatomical structures. It is thought that the best three-dimensional position calls for the same long axis of the implant as the tooth inside the alveolar bone. When the angle was > 25°, the length from the apex to the palatal plane along the long axis of the tooth was < 6 mm (Table 6), indicating a higher likelihood of obtaining sufficient primary implant stability when placing the implant along the long axis of the tooth.

### Implications and future prospects

#### Implications for using the sagittal root angle as an index for immediate implant placement

From the results described above, we can see that a smaller sagittal root angle corresponded to thinner buccal and thicker palatal bone around both the CEJ and apex, and more available bone volume under the root apex along the tooth axis. A scatter diagram and regression equation of the sagittal root angle and these correlated indices have been created to quantitatively describe how the angle affects the alveolar bone distribution. This made it possible to use the root angle to predict the value of other implant-related anatomical indices, which further proved that the angle index should play a more important role in the immediate implant evaluation system as a whole. Based on the characteristics of these implant-related index values according to different angle groupings (Table 7), we attempted to divide the root angle into four types to better utilize the angle index for immediate implant placement.

**Table 7:** Four types of anatomical characteristics of maxillary anterior teeth for immediate implant according to the sagittal root angle with surrounding hard and soft tissue

Anatomical type	Angle	n	Index		Result	Significance	Implication for immediate implant design	Schematic map of implant(Bone level NC, Straumann; 3.3*12 mm)
Type I	< 0°	9	Coronary buccal bone and gingiva	Bone	< 1 mm	High risk of buccal bone wall defect when placing the implant	The implant could be place along the axis of tooth, while the palatal bone Under the CEJ should be noted to keep contact (> 1 mm at least)	
				Gingiva	No correlation	/		
			Coronary palatal bone		1.5–1.7 mm	Relative thin for offering alternative bone		
			Apical bone	Labial	1.0–3.0 mm	Ensuring sufficient primary stability for immediate implant		
				Palatal	2.0–4.0 mm	Relative thick for offering alternative bone		
			Under apex bone along the tooth axis		8.4 mm	To get enough primary stability		
			Root diameters		No correlation	/		
Type II	0°–10°	125	Coronary buccal bone and gingiva	Bone	0.51–1.20 mm	High risk of buccal bone wall defect when placing the implant	The implant could be place along the axis of tooth, which was the best anatomic situation (not only the angle, but the surrounding bone) for immediate implantation	
				Gingiva	No correlation	/		
			Coronary palatal bone		1.6–2.2 mm	Relative thick that could be used to adjust implant to proper position		
			Apical bone	Labial	1.0–3.0 mm	Ensuring sufficient primary stability for immediate implant		
				Palatal	2.3–5.1 mm	Thick for offering alternative bone		
			Under apex bone along the tooth axis		9.9–10.1 mm	To get enough primary stability		
			Root diameters		No correlation	/		
Type III	10°–25°	545	Coronary buccal bone and gingiva	Bone	0.50–1.03 mm	High risk of buccal bone wall defect when placing the implant	The implant was recommended to rotate palatally at the apical direction to protect the buccal bone wall around the apex. Intro-mouth adhesive of the crown and even angled implant base might be used during final restoration	
				Gingiva	No correlation	/		
			Coronary palatal bone		1.7–3.1 mm	Adequate bone dimension that could be used to adjust implant to proper position		
			Apical bone	Labial	0.9–2.1 mm	< 1 mm usually indicates high risk of fenestration and perforation during implant placement		
				Palatal	3.1–8.1 mm	/		
			Under apex bone along the tooth axis		6.4–9.4 mm	Abundant bone for getting enough primary stability		
			Root diameters		No correlation	/		
Type IV	> 25°	121	Coronary buccal bone and gingiva	Bone	< 1 mm	High risk of buccal bone wall defect when placing the implant	The implant has to rotate palatally to protect the buccal bone wall. Angled implant base would be used during final restoration or immediate implant was not recommended on the consideration of long-term success	
				Gingiva	No correlation			
			Coronary palatal bone		2.2–4.3 mm	Adequate bone dimension that could be used to adjust implant to proper position.Adequate bone dimension that could be used to adjust implant to proper position		
			Apical bone	Labial	0.8–1.4 mm	< 1 mm usually indicates high risk of fenestration and perforation during implant placement		
				Palatal	4.4–10.3 mm	/		
			Under apex bone along the tooth axis		2.9–4.6 mm	High risk to get enough primary stability		
			Root diameters		No correlation	/		

For type I, the angle was < 0°. The coronal buccal and palatal bone was thin, but the bone mass around and under the root apex along the long axis of the tooth were rather abundant. When planning an immediate implant, the implant could be inserted along the long axis of the tooth to achieve sufficient primary implant stability, and more attention should be paid to the coronal bone to ensure intact palatal bone wall.

For type II, the angle was 0°–10°. The coronal labial and palatal bone were relatively thicker, and the bone mass around and under the root apex along the long axis of the tooth were rather abundant. It was a satisfactory anatomical situation for immediate implant placement along the tooth axis with sufficient primary implant stability and contact with the labial and palatal bone.

For type III, the angle was at 10°–25°. The coronal labial and palatal bone were relatively thick, and the bone mass under the root apex was rather abundant, but the thickness of the buccal bone at levels 2 mm and 4 mm coronal to the apex was < 1 mm. This indicated a higher risk of fenestration and perforation at the apical labial side when placing an implant along the tooth extraction socket. To protect the labial wall, the implant should be placed along the palatal wall in the extraction socket without touching the facial bone wall.

For type IV, the angle was > 25°. Although the coronal palatal and apical palatal bone were thick, the coronal labial bone and apical labial bone were < 1 mm, and the bone thickness under the root apex along the long axis of the tooth was < 6 mm. This anatomical situation made it extremely difficult for the clinician to perform an immediate implant, as not only was there a high risk of fenestration and perforation at the buccal bone wall, but there was also poor primary implant stability when placing the implant along the axis of the tooth extraction socket. It would be recommended to translate and rotate the implant placement to the palatal side to reduce the angle between the implant and the alveolar bone, and to be more consistent with the long axis of tooth that has abundant bone under the root apex. An angled implant base would be used during the final restoration to ensure stability. Nevertheless, using another restoration method would be a more compromised, but sensible, option. It should be noted, that no matter what the angle was, the coronal buccal bone was thin (< 1.2 mm), which had high risk of bone absorbing after tooth extraction gradually. To obtain optimal esthetic outcome, buccal bone augmentation was always recommended when placing an implant in the presence of concerns for long-term esthetics of immediate implant.

### Implications of using the sagittal root angle for immediate implant patient selection

The sagittal root angle as measured in the present study was primarily in a range of 10°–25° in the study population, and the average angle was also in the range of 10°–25°, which indicted that for most patients, there was relatively high risk of fenestration and perforation at the buccal bone wall when performing immediate implant placement in the anterior teeth. As mentioned above, there was a significant difference in the root angle, nearly 4°, between the lateral and central incisors. The angle in males was nearly 2° larger than that in females, and there was a nearly 3° increase in the angle in the 18–30, 30–50, and > 50 years age groups. Based on the results found in the study population, younger females with complaints regarding the lateral incisor had the highest possibility of presenting with a minor root angle and other implant-related anatomical features of the alveolar bone sufficient for immediate implant placement in the anterior teeth. In contrast, older males with complaints regarding the central incisor have the highest possibility of presenting with an increased root angle and relatively poor anatomical features of the alveolar bone, which are inadequate for immediate implant placement in the anterior teeth, and require additional attention.

It should be noted that due to the limitations of the plaster models, only 130 teeth were included for gingiva measurement and analysis, which may have affected the accuracy of the conclusion that the gingiva has no correlation with the sagittal root angle. Although the scatter diagram and regression equation for the root angle and its correlated indices have been created based on the expectation of using the angle to directly calculate other related indices, the interpretability of the regression equation was not satisfactory, with a coefficient of determination < 0.4. The quantifiable relationship between the sagittal root angle and these correlated implant-related anatomical indices was complicated, and additional studies involving more professional statistical methods and larger sample sizes are needed to further evaluate the effects of the angle as an index for the evaluation of immediate implant placement.

With the development of digital technology [30, 31], the accuracy of computer-guided template-assisted implant surgery and computer-guided sleeve-designed template has been confirmed [32–34]. Via carefully pre-design, guided surgery can improve the treatment outcomes in patients with critical soft and hard tissue conditions. This makes guided surgery very helpful especially in patients with large sagittal root angles, which requires the high accuracy in execution of implants and were considered high technical sensitive. This study provided thorough understanding of sagittal root angle, which is helpful for clinician to assess the difficulties of immediate implant and determine the need of applying guided surgery in pre-operative design and implant placement.

## Supplementary Information

**Additional file 1. Fig. S1**: Standardized protocols were used to obtain the sagittal sections to be used for measurement. (a) The red line shows the tooth axis (TA) in the sagittal plane, crossing the incisor and apical points. The white line shows the corresponding alveolar bone axis (BA). The angle between TA and BA (TA-BA) was measured in degrees. (b)The white dotted line is parallel to CEJ, and the number after C represents the vertical distance to CEJ. The red line segment represents the corresponding soft tissue thickness, and the green line segment represents the thickness of the labial bone. There is only soft tissue thickness information on C-0 line. (c)The white dotted line is parallel to CEJ, and the number after C represents the vertical distance to CEJ. The green line segment represents the corresponding thickness of the palatal bone. (d)The R-0 line is parallel to the CEJ and passes through the apical point, and the R-2 and R-4 lines are parallel to the R-0 line and are separated by 2mm and 4mm, respectively. The green part represents the thickness of the labial and palatal wall. (e)Green lines represent bone dimensions below the apex, including the length of the root apex to the alveolar palatal plane((TA-or-BA-Apex to Palate), measured along the long axis of the anterior tooth (CE line) and the corresponding alveolar bone axis( DF line). (f)The white dotted line represents the line parallel to CEJ. The yellow ones represent the root dimension.

**Additional file 2. Fig. S2**. The process of entering DICOM and STL files into Adobe Illustrator Software to get standard screenshots with hard tissue and soft tissue information.

## Data Availability

The datasets generated and analysed during the current study are available from the corresponding author on reasonable request.
