# Supplementary material for: Analysis of the sagittal root angle and its correlation with hard and soft tissue indices in anterior teeth for immediate implant evaluation: a retrospective study
Source: BMC Oral Health. 2021 Oct 4;21:494. doi: 10.1186/s12903-021-01848-x (PMC8491410; doi:10.1186/s12903-021-01848-x)
Supplement: Supplementary file 1 — Additional file 1. Fig. S1: Standardized protocols were used to obtain the sagittal sections to be used for measurement. (a) The red line shows the tooth axis (TA) in the sagittal plane, crossing the incisor and apical points. The white line shows the corresponding alveolar bone axis (BA). The angle between TA and BA (TA-BA) was measured in degrees. (b)The white dotted line is parallel to CEJ, and the number after C represents the vertical distance to CEJ. The red line segment represents the corresponding soft tissue thickness, and the green line segment represents the thickness of the labial bone. There is only soft tissue thickness information on C-0 line. (c)The white dotted line is parallel to CEJ, and the number after C represents the vertical distance to CEJ. The green line segment represents the corresponding thickness of the palatal bone. (d)The R-0 line is parallel to the CEJ and passes through the apical point, and the R-2 and R-4 lines are parallel to the R-0 line and are separated by 2mm and 4mm, respectively. The green part represents the thickness of the labial and palatal wall. (e)Green lines represent bone dimensions below the apex, including the length of the root apex to the alveolar palatal plane((TA-or-BA-Apex to Palate), measured along the long axis of the anterior tooth (CE line) and the corresponding alveolar bone axis( DF line). (f)The white dotted line represents the line parallel to CEJ. The yellow ones represent the root dimension. [file 12903_2021_1848_MOESM1_ESM.docx]

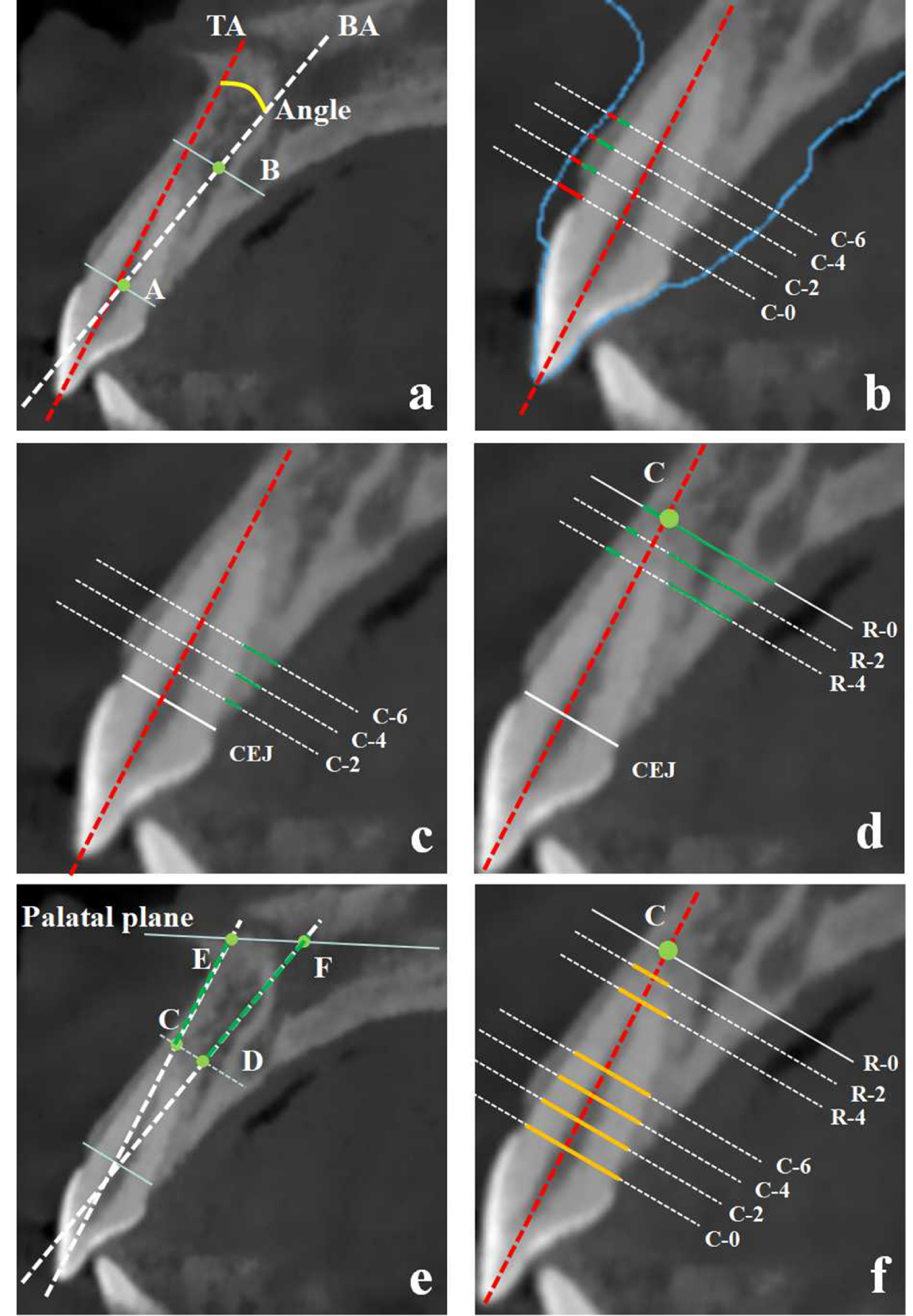


**Supplementary Figure 1.** Standardized protocols were used to obtain the sagittal sections to be used for measurement. (a) The red line shows the tooth axis (TA) in the sagittal plane, crossing the incisor and apical points. The white line shows the corresponding alveolar bone axis (BA). The angle between TA and BA (TA-BA) was measured in degrees. (b)The white dotted line is parallel to CEJ, and the number after C represents the vertical distance to CEJ. The red line segment represents the corresponding soft tissue thickness, and the green line segment represents the thickness of the labial bone. There is only soft tissue thickness information on C-0 line. (c)The white dotted line is parallel to CEJ, and the number after C represents the vertical distance to CEJ. The green line segment represents the corresponding thickness of the palatal bone. (d)The R-0 line is parallel to the CEJ and passes through the apical point, and the R-2 and R-4 lines are parallel to the R-0 line and are separated by 2mm and 4mm, respectively. The green part represents the thickness of the labial and palatal wall. (e)Green lines represent bone dimensions below the apex, including the length of the root apex to the alveolar palatal plane((TA-or-BA-Apex to Palate), measured along the long axis of the anterior tooth (CE line) and the corresponding alveolar bone axis( DF line). (f)The white dotted line represents the line parallel to CEJ. The yellow ones represent the root dimension.
