# Supplementary material for: Analysis of the sagittal root angle and its correlation with hard and soft tissue indices in anterior teeth for immediate implant evaluation: a retrospective study
Source: BMC Oral Health. 2021 Oct 4;21:494. doi: 10.1186/s12903-021-01848-x (PMC8491410; doi:10.1186/s12903-021-01848-x)
Supplement: Supplementary file 2 — Additional file 2. Fig. S2. The process of entering DICOM and STL files into Adobe Illustrator Software to get standard screenshots with hard tissue and soft tissue information. [file 12903_2021_1848_MOESM2_ESM.docx]

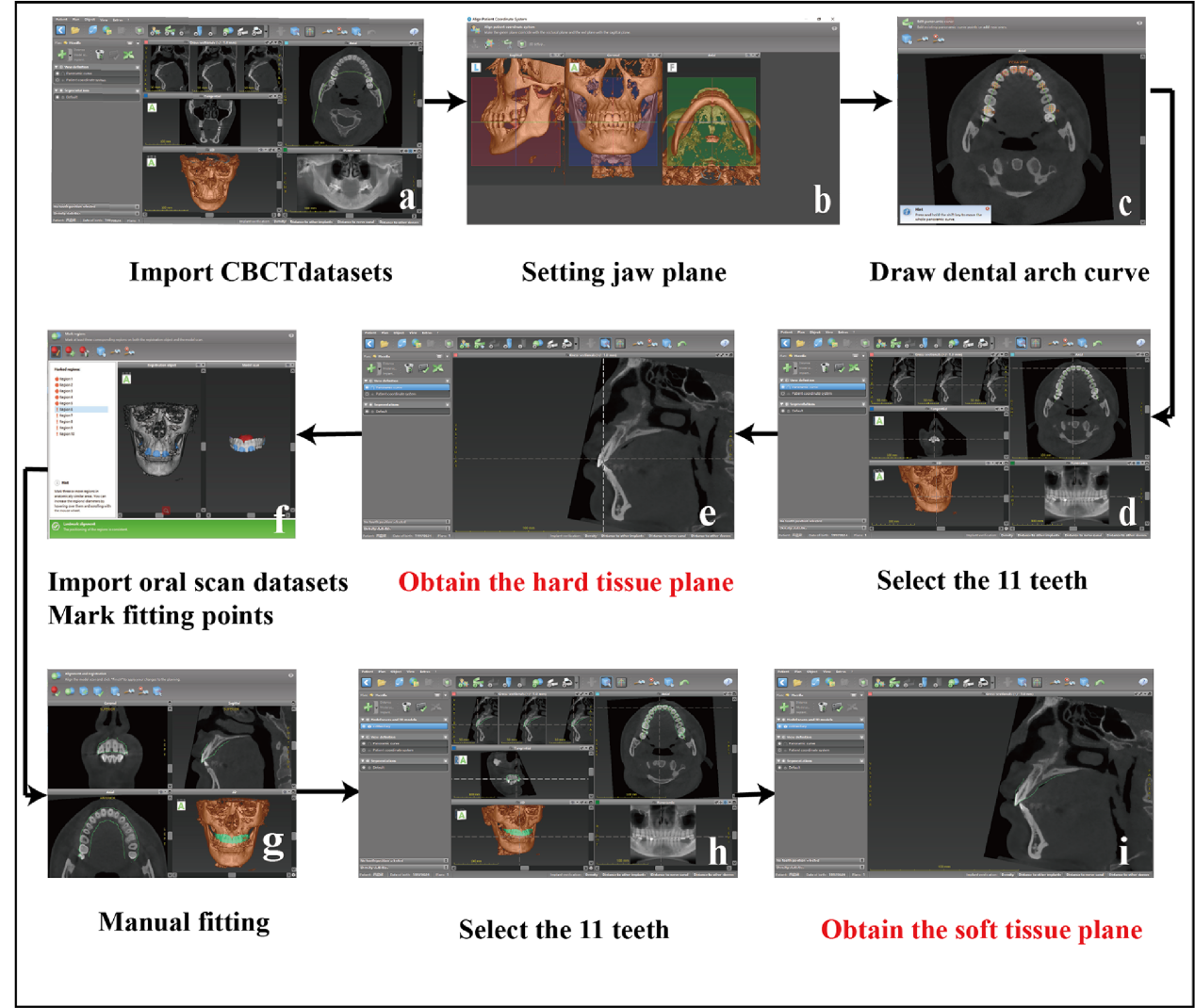


**Supplementary Figure 2.** Enter DICOM and STL files into Adobe Illustrator Software to get standard screenshots with hard tissue and soft tissue information.
